# Predictors of Static Postural Loading in Primary-School-Aged Children: Comparing Elastic Net and Multiple Regression Methods

**DOI:** 10.3390/children12060744

**Published:** 2025-06-08

**Authors:** Mohammad Ali Mohseni Bandpei, Reza Osqueizadeh, Hamidreza Goudarzi, Nahid Rahmani, Abbas Ebadi

**Affiliations:** 1 Neuromusculoskeletal Rehabilitation Research Center, University of Social Welfare and Rehabilitation Sciences, Tehran 1985713871, Iran; mohseni_bandpei@uswr.ac.ir; 2 Pediatric Neurorehabilitation Research Center, University of Social Welfare and Rehabilitation Sciences, Tehran 1985713871, Iran; 3 Department of Mathematics, Faculty of Science, Yasouj University, Yasouj 7591874831, Iran; goudarzi@mail.yu.ac.ir; 4 Department of Physiotherapy, University of Social Welfare and Rehabilitation Sciences, Tehran 1985713871, Iran; na.rahmani@uswr.ac.ir; 5 Nursing Care Research Center, Clinical Sciences Institute, Baqiyatallah University of Medical Sciences, Tehran 1435915371, Iran; ebadi1347@bmsu.ac.ir

**Keywords:** children, posture, sedentary behavior, regression

## Abstract

**Background/Objectives:** Adverse effects of a sedentary lifestyle on an individual’s overall health are inevitable. With reference to primary-school-aged children, the establishment of effective postural hygiene is critical as it not only promotes optimal musculoskeletal development but also significantly influences their long-term well-being and productivity. This study aimed to develop and internally validate a regularized regression model to predict static postural loading (SPL) in primary school children. **Methods:** The outcome and predictors of SPL were shortlisted through a systematic review of the literature and expert panels. Data were derived from 258 primary school children. We developed regularized elastic net (EN) and used multiple linear regression (MLR) as a reference. Both models were fitted through five-fold cross-validation with 10 iterations. The grid search technique was used to find the optimal combination of hyperparameters α and λ for the EN. We conducted a permutation importance analysis to obtain and compare predictor rankings for each model. **Results:** Both models presented a good and comparable fit, with the EN marginally outperforming the MLR in error metrics. Postural risk, sedentary behavior, task duration, and BMI were the most important predictors of SPL in primary school children. **Conclusions:** The proof of a direct impact of a sedentary lifestyle on children’s overall health is both credible and alarming. Hence, proper identification and management of contributing factors to static postural loading in this age group is critical. In various clinical settings, where the objective is to develop a model that accurately forecasts the outcome, advanced regularized regression methods have evidently shown great performance.

## 1. Introduction

The global population of primary-school-aged children reached an all-time high of 820 million in 2023 and is projected to peak once more at approximately 806 million in the early 2050s [1]. The significance of postural health in this age category cannot be overstated as it ensures their long-term health and productivity. Fundamental physical changes [2,3], the formation of spinal curves [4,5], puberty [6,7], and a continuously growing sedentary lifestyle [8,9,10] severely impact postural habits and physical health in primary-school-aged children. In a cross-sectional study conducted in 2021, Santos reported a 27% prevalence of back pain in children aged 6 to 12 years, related to long hours of screen time and inappropriate designs of backpacks. Hence, a lack of monitoring and strict control measures in this regard increases the possibility of various musculoskeletal issues in the short term [11,12,13] and long term [14] among children as future human resources.

Consequently, significant direct and indirect costs are imposed on families and governments [15,16,17,18,19,20]. The results of a ten-year cohort study in Canada indicated a 51% difference in treatment costs between two groups of people with and without musculoskeletal disorders [21]. In addition, a recent study in Europe revealed an average of 2% of gross domestic product expenditure on similar issues [22]. Therefore, commitment to implementing primordial and primary public health prevention strategies [23] highlights the importance of reviewing the factors associated with physical workload to properly design products, tasks, and environments for this age group [24,25].

Factors predicting static postural loading (SPL) have been broadly investigated in several studies. These mainly include personal, physical, and task-related parameters. In this regard, a wide variety of accurate and efficient regression modeling methods have been employed to quantify the physical workload that is being imposed on an individual’s body, mainly comprising correlation studies [26,27], standard linear regression models [28,29], and logistic regression models [30,31,32].

As regards SPL in children that are subject to diverse biomechanical, physiological, and environmental parameters, the presence of multicollinearity may arise due to high correlations among predicting variables. This can lead to potential bias and variance in parameter estimates, ultimately resulting in inaccurate evaluations. In this regard, regularized regression methods offer practical solutions, specifically in handling issues such as multicollinearity and overfitting. By incorporating a penalty term into the loss function, these methods effectively constrain the coefficients of the model, promoting simpler and more interpretable results. This regularization not only enhances the model’s generalization capabilities on unseen data but also allows for better feature selection, as irrelevant variables can be effectively shrunk towards zero, resulting in a simple and more reliable predictive model [33].

The current study was designed to investigate the factors associated with physical workload among primary school children while performing sedentary tasks. More specifically, it aimed to predict SPL in this age group via classic and advanced regression modeling techniques. For this purpose, multiple linear regression (MLR) and regularized elastic net (EN) models were applied on a dataset derived from primary school children. The underlying hypothesis was that combining these two approaches would enable precise weighting of the potential factors previously identified in the literature. Another assumption was that the EN would partially outperform MLR in predicting SPL in this age group.

## 2. Materials and Methods

### 2.1. Study Design

**Figure 1:**
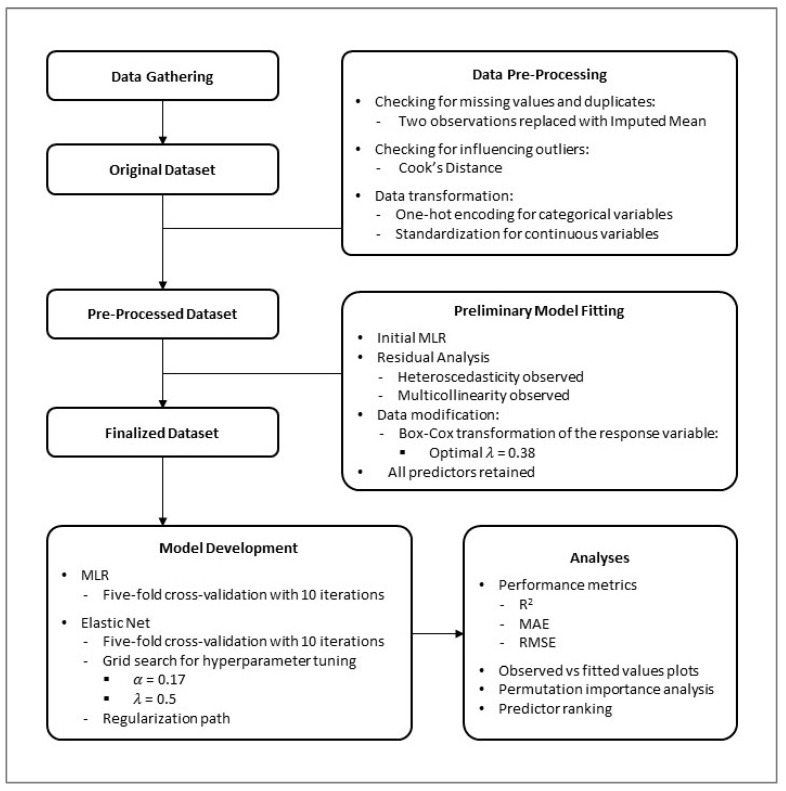
The procedural steps of the study.

This study employs an analytical cross-sectional design, with data gathered from primary school children in Tehran between February and June 2023. We developed and internally validated the EN regression model to predict SPL in this age group. The detailed procedural steps of the study are summarized in Figure 1.

### 2.2. Population

Children enrolled in any of the six grades of primary school were eligible for participation in the study. Although the primary focus of the research did not include gender and school grade, the sample was stratified by these two parameters to enhance the comprehensiveness of the population representation. We excluded those with significant musculoskeletal issues or any physical diseases/conditions (including records of spinal or limb surgeries) likely to influence physical workload assessments via a preliminary screening process.

### 2.3. Outcome

The primary outcome was to develop a regression model that accurately predicts postural loading in primary school children while engaged in sedentary tasks. The Cornell Musculoskeletal Discomfort Questionnaire (CMDQ) was utilized as a feasible tool to evaluate body posture and comfort [34,35,36]. Accordingly, the overall computed score of this tool was considered as a reference for the response variable in the current study.

### 2.4. Candidate Predicting Variables

Potential predicting variables were shortlisted through a systematic literature review [37] and expert panels with clinicians and academics in the field. Of these variables, ten entered the model development stage. The information was primarily based on self-reported questionnaires, direct observations, and clinical measurements. As regards personal factors, in addition to age [38] and gender [39], we evaluated participants’ physical inactivity [40,41] via the Sedentary Behavior Questionnaire (SBQ) [42]. As children grow, their physical and cognitive development progresses, affecting their postural control and ability to maintain proper alignment during sedentary activities. Younger children may exhibit less developed postural awareness and control, making them more susceptible to poor posture during prolonged sitting. Gender differences further complicate this dynamic; for instance, boys and girls may differ in body composition, activity levels, and postural habits, which can influence how they respond to sedentary tasks. Additionally, sedentary behavior has emerged as a significant concern as increased screen time and prolonged sitting are associated with adverse health outcomes, including poor posture and musculoskeletal discomfort.

Physical characteristics, including body mass index (BMI) [43,44] and body fat percentage [45,46], were estimated based on the four-site skinfold Westrate equation [47]. Musculoskeletal health [48,49] of the participants was assessed by a certified specialist using the pediatric version of the GALS (Gait, Arms, Legs, and Spine) examination tool [50]. BMI serves as a widely used indicator of body weight relative to height, providing insights into whether a child is classified as underweight, healthy, overweight, or obese. Elevated BMI often correlates with an increased body fat percentage, which can lead to excess strain on the musculoskeletal system, particularly during sedentary activities. Children with higher body fat percentages may experience greater challenges in maintaining proper posture due to altered body mechanics and increased fatigue. Furthermore, musculoskeletal health is crucial for maintaining optimal posture; children with pre-existing musculoskeletal issues or weaknesses may be more prone to adopting maladaptive postures, exacerbating the risks associated with prolonged sitting.

In terms of task-related factors, we observed children’s prominent sitting type [51] and evaluated anthropometric match [52] with the utilized furniture according to standard equations [53]. In addition, sedentary task duration [54,55] was recorded, and postural risk [56] was estimated using the Rapid Entire Body Assessment (REBA) [57]. Different sitting types influence spinal alignment and overall comfort during prolonged periods of sitting. The anthropometric match between a child’s body dimensions and the design of their furniture is essential; an appropriate fit promotes correct posture and reduces the likelihood of musculoskeletal strain. Additionally, the duration of sedentary tasks plays a vital role in postural health; extended sitting periods can lead to cumulative postural fatigue and discomfort, increasing the risk of developing poor postural habits. In addition, the REBA postural risk score provides a systematic approach to assessing the risk associated with specific postures during tasks, helping to identify individuals at higher risk for posture-related issues.

Given the study’s expected scientific application, genetic risk factors and biomarkers were not taken into account as potential contributors. The complete list of candidate predicting variables is presented in Table S1.

### 2.5. Statistical Analysis

#### 2.5.1. Data Pre-Processing

The original dataset underwent detailed pre-processing to improve data quality. First, Cook’s distance was consulted to detect any influencing outliers (Figure S1). We addressed the presence of two missing observations $\left(7\times 10^{-4}\%\right)$ in our dataset through mean imputation. In addition to its simplicity and effectiveness, the absence of influencing outliers in our dataset supported the appropriateness of mean imputation as it reduced the risk of skewing the results. To further validate our choice, we conducted a sensitivity analysis, which demonstrated that the results remained consistent across various imputation scenarios (Table S1). Next, we transformed data through encoding and scaling. The one-hot encoding technique was applied to convert categorical predicting variables into numerical values, and standardization was utilized to match the scales of all continuous predicting variables. Residual analysis via an initial multiple regression revealed degrees of heteroscedasticity and multicollinearity. Accordingly, the Box–Cox transformation of the response variable was carried out with an optimal λ value of 0.368. We opted not to consider the Variance Inflation Factor (VIF) primarily to maintain the integrity of our model by including all predictor variables, particularly three categorical variables with multiple levels. The nature of the research necessitated a comprehensive approach, as excluding any variable based on VIF thresholds could have resulted in the omission of significant predictors [58]. This would undermine the overall comprehensiveness of the model. EN regression is particularly advantageous in this context as it effectively addresses multicollinearity through its inherent regularization techniques. Moreover, EN’s flexibility in handling various types of predictors, including continuous and categorical variables, makes it particularly suitable for complex datasets. Its ability to automatically adjust the degree of regularization based on the data characteristics ensures that the model remains robust against overfitting. This is crucial in predictive modeling, where the goal is not just to fit the training data but also to generalize well to unseen data.

#### 2.5.2. Model Fitting and Evaluation

We developed regularized EN and MLR models to predict SPL based on the same set of predicting variables. The MLR model was fitted as a baseline for evaluations and specified using ordinary least squares (OLS). In the case of the EN, the OLS estimation was modified by adding L1 (LASSO) and L2 (Ridge) penalty terms to the loss function [59]. The regularization in the EN relied on hyperparameter α to establish the overall strength of the regularization applied to the model and hyperparameter λ to manage the balance between L1 and L2 penalties. A description of the EN model is presented in Appendix S1.

Both models were implemented through five-fold cross-validation with 10 iterations, resulting in a total of 50 training and validation cycles. Performance metrics, including R2, mean absolute error (MAE), and root mean squared error (RMSE) were averaged over all cycles for both models. Hyperparameter tuning for the EN was preformed using the grid search method. For α, we selected values of [0.1, 0.5, 0.9] to explore a range of mixing ratios between L1 and L2 regularization, allowing for flexibility in the model’s complexity. In addition, values of [10^−4^, 10^−3^, 10^−2^, 10^−1^, 1, 10^1^, 10^2^] were considered for λ to be inclusive for the EN model’s regularization strength. The grid search evaluated a total of 7×3=21 combinations, which yielded optimal values of α (0.17) and λ (0.5). The regularization path for the EN model is displayed in Figure S3.

We conducted permutation importance analysis to identify the most influential predictors for both models. The approach is based on the concept of assessing the impact of individual variables on a model’s predictive performance by evaluating how shuffling the values of a variable affects the model’s accuracy [60]. All analyses were performed in Google Colab environment using Python Programming Language (Version 3-12-2) [61].

## 3. Results

The study population comprised 258 participants, with a mean age of 9.4 years (SD = 1.7). In terms of physical characteristics, participants had an average BMI of 16.9 (SD = 4.8), with values spanning from 7.5 to 28.6 kg/m^2^. As regards musculoskeletal examinations, 157 participants (39.1%) had conditions affecting their physical health. The study also evaluated sedentary task-related factors. Among the participants, 28.7% adopted an upright sitting position, while the majority exhibited improper postures, including slumped, slouched, and leaning forward postures during sedentary activities. However, the assessment of postural risk yielded an acceptable mean score of 3 (SD = 1.2). Meanwhile, anthropometric measurements revealed that 53.5% of the participants experienced mismatch in relation to the utilized sitting furniture dimensions. Table 1 provides the participants’ data and the results of the sedentary task-related measures.

In this study we compared the fit and performance of the MLR and EN models. The R2 values observed for both models were high and identical. For both models, the R2 value decreased when evaluated on the test dataset compared to the training dataset. Looking at error metrics, the EN model appeared to have a slight edge compared to MLR as it had a lower MAE in the test dataset (2.452 vs. 2.494). Likewise, the EN model achieved a marginally lower RMSE of 3.199 compared to 3.202 for the MLR. For both models, the MAE and RMSE values increased when comparing across training and test datasets. The results of the model performance comparisons are presented in Table 2.

Table 3 summarizes the results of permutation variable importance analysis for the models, providing insights into the contribution of each variable to the overall model performance. Postural risk, BMI, task duration, and sedentary behavior were the most important factors affecting both models’ performance in predicting SPL among primary school children. The corresponding variable importance charts are presented in Figure S4.

## 4. Discussion

Our study contributes to physical health knowledge in two manners. First, it presents an organized review of the factors related to the concept of physical workload during sedentary tasks, which is theoretically applicable in providing proper postural hygiene for the given population. Second, it delivers the results of a comparison between the performance of regularized EN and MLR models in predicting static postural loading on a dataset derived from primary school children.

### 4.1. Main Predictors of SPL in School Children

The alignment of body segments plays a crucial role in determining how forces are distributed across the musculoskeletal system during static activities. This observation underscores why postural risk in our research was identified as the most significant factor in predicting static physical workload, as demonstrated by MLR and EN regression models. When body segments are misaligned, it leads to increased stress on specific joints and muscles, thereby elevating the overall experienced musculoskeletal discomfort. This issue has also been addressed in previous research [62,63,64]. In a recent study, an association between maladaptive postures in the neck and upper extremity and the development of musculoskeletal symptoms was highlighted [65]. We used a dual approach to measure posture in this study. First, we used a semi-quantitative and widely accepted observational tool (REBA), which yielded a predictor of SPL with the highest permutated importance ranking in both the MLR (0.208 (SD = 0.047)) and EN (0.118 (SD = 0.032)) regression models. Second, we conducted a direct observation of sitting posture that resulted in a low-impact categorical predictor of SPL in the models. Our findings, along with previous research, indicate the importance of postural hygiene and the quantification of posture in controlling the physical load imposed on children’s musculoskeletal systems while being engaged in sedentary tasks.

Our research findings indicate that sedentary behavior and body mass index (BMI) are significant contributors to the response variable examined in this study. While it would be incorrect to assert a direct correlation between an individual’s physical inactivity and their experienced local physical workload, it is possible to clarify an indirect relationship by examining the role of BMI as an intermediary factor. The impact of a sedentary lifestyle on children’s obesity has been highlighted in several studies [66,67,68,69]. Respectively, a wide variety of preventative measures to promote physical activity among school-aged children have been explored. Behavioral and environmental interventions in school and family settings [70,71,72], designing compatible classroom furniture [73], and objectively measuring children’s sedentary behavior and physical activity [74,75,76] are approaches of particular interest.

Surprisingly, anthropometric match with school furniture was considered less influential in estimating physical workload among children by our models. Nonetheless, the association between school furniture design and musculoskeletal discomfort is well documented in the literature [52,54,77]. In a comprehensive systematic review, Castelluchi et al. provide details of equations to accurately evaluate any mismatches between school furniture and students’ body dimensions [53]. These measures were also consulted for anthropometric assessments in our study. As one would expect, a proper anthropometric match and the standard adjustment of classroom furniture directly affect the posture that is adopted by individuals while interacting with such products. Children may have varying levels of adaptability to different furniture sizes and shapes. Some may compensate for a poor anthropometric match with postural adjustments that mitigate potential discomfort or strain, which could mask the expected relationship. In addition, the methods used to assess static postural loading and anthropometric match may have limitations. If the measurements were not sensitive enough to capture subtle differences, this could result in an underestimation of the impact of anthropometric match.

Task duration was another influential factor identified by the regression models in our study. Previous research indicates a meaningful association between sedentary task duration and the amount of muscular load that is experienced by children [54,55], thus affecting their overall health and well-being [72]. By definition, specific types of muscular activity and inadequate recovery after local fatigue interact and contribute to the occurrence of muscular discomfort during static physical work [34]. Challenges in resisting gravity and other external forces to stabilize and maintain the required body posture in sedentary tasks is another underlying biomechanical factor to justify the significance of proper timing for such activities.

To assess the significance of predictors, we employed permutation importance analysis, which allowed us to evaluate and compare the importance of predictors both within and between the two models. This method provides valuable insights into how each predictor contributed to model performance, reinforcing the necessity of understanding predictor significance in model evaluation and selection. Overall, our findings highlight the importance of contextual factors in model performance and the utility of permutation importance in guiding interpretative insights.

### 4.2. Regression Models

**Figure 2:**
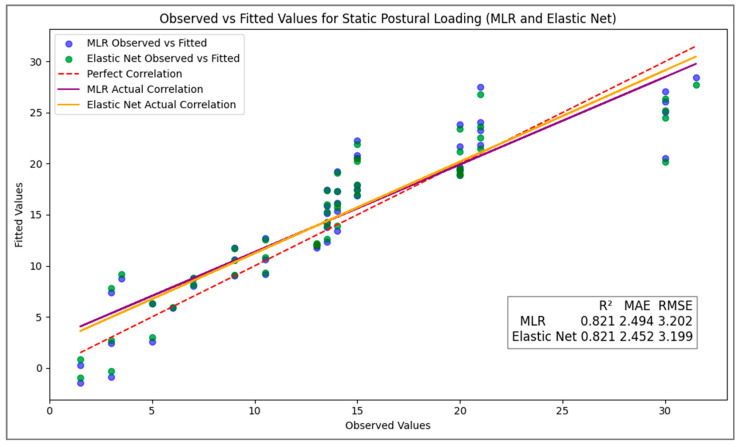
Observed vs. fitted values. The plot was generated using the Matplotlib library in Google Colab environment using Python Programming Language (Version 3-12-2).

In our study, we implemented MLR and EN regression on the same dataset to evaluate their performance in predicting the response variable. As evident in Figure 2, both models demonstrated strong predictive capabilities, with MLR providing a robust baseline due to its simplicity and interpretability. However, the EN regression model exhibited a slight edge over MLR in terms of error metrics, suggesting that it was more effective in capturing the underlying patterns within the data.

Interestingly, our research indicates that regularization did not significantly enhance the performance of the EN compared to MLR. This finding suggests that, in this particular context, the benefits of regularization, typically associated with improved model generalization and feature selection, were not as expected. The marginal advantage of EN over MLR may imply that the dataset’s characteristics and the relationships among predictors did not necessitate the complexities introduced by regularization.

One of the primary advantages of MLR is its simplicity and ease of interpretability. In contrast, the complexity introduced by the EN model, while beneficial for addressing multicollinearity and enhancing predictive power, may conceal the interpretability of individual predictors. Furthermore, the marginal performance improvement in the EN model may not be substantial enough to warrant the additional complexity it introduces. In scenarios where the primary goal is to provide interpretable insights rather than to maximize predictive accuracy, MLR may be the preferred choice. For instance, in fields such as healthcare, where understanding the impact of individual variables is critical, the straightforward nature of MLR can facilitate more effective communication and applications of findings.

However, there are specific scenarios where the benefits of the EN model become particularly relevant. In complex datasets with numerous predictors, the EN model’s ability to perform variable selection and regularization can lead to more stable and reliable predictions. In such cases, the marginal improvement in predictive performance may be more noticeable, justifying the use of the EN model despite its complexity. Additionally, when the primary objective is to develop a robust predictive model for deployment in real-world applications, the EN model may provide a competitive advantage. Its enhanced ability to generalize to unseen data can lead to improved outcomes in predictive tasks. In these contexts, the trade-off between interpretability and predictive performance may be acceptable as the ultimate goal is to achieve superior predictive accuracy.

### 4.3. Implications

Educators play a pivotal role in shaping children’s postural habits. The findings suggest that prolonged task duration may contribute to increased SPL, indicating a need for structured breaks during classroom activities. Educators should be encouraged to implement short, frequent breaks that allow children to engage in physical activity, thereby reducing static loading and promoting better postural alignment. Additionally, integrating postural education into the curriculum can raise awareness among children about the importance of maintaining proper posture during various tasks.

Similarly, clinicians working with children, particularly those in pediatric healthcare and rehabilitation, should consider integrating the identified predictors into routine assessments. By routinely evaluating postural risk factors, clinicians can develop tailored intervention strategies that address the specific needs of each child. Furthermore, clinicians should advocate regular screenings of SPL during routine health check-ups in schools, allowing for the early identification of at-risk children. This proactive approach can facilitate timely interventions, potentially reducing the incidence of musculoskeletal issues related to poor posture.

Equally important, policymakers should consider the implications of these findings when developing guidelines and standards for physical education and health in schools. Establishing policies that mandate regular physical activity and clinical assessments in school settings can foster an environment that prioritizes postural health. Moreover, incorporating SPL assessments into national health initiatives can help track trends in children’s postural health, informing future policy decisions and funding allocations. Collaborative efforts among healthcare providers, educators, and policymakers are vital in creating comprehensive strategies aimed at reducing SPL and improving overall postural health in children.

### 4.4. Strengths and Limitations

To the best of our knowledge, this research represents one of the first few studies to develop and internally validate the EN regression model in the context of our dataset, providing useful insights into the performance and applicability of regularized methods. In addition, we followed the recommended procedures in developing and evaluating the EN model, ensuring precision in our methodology and enhancing the reliability of our findings.

There are also limitations to consider. Due to the limited availability of suitable external databases that align with our study parameters, we only performed internal validation. Therefore, although rigorous cross-validation and hyperparameter tuning are intended to enhance our model’s performance on unseen data, the model may still lack generalizability. In addition, due to the cross-sectional nature of the study, the findings are based on a sample of specific children at the time of the analysis. So, population dynamics and emerging factors over time should be considered when interpreting the results. Also, we did not explore other advanced regression methods, including Bayesian Regression, Gradient Boosting Machines, and Support Vector Regression. In addition, while the mean imputation technique that we employed to address the missing observations was an appropriate choice for our dataset, it is essential to acknowledge its limitations, such as its potential to underestimate variability and the risk of masking relationships between variables.

In addition, the marginal improvement in the EN over MLR raises questions about the necessity of regularization in certain scenarios, suggesting that further research is required to explore the conditions under which regularized models provide significant advantages. Additionally, we retained all the important predictors identified in the literature, which may disrupt the performance of the MLR model in terms of multicollinearity. Future research in this context should aim to replicate this analysis across large and diverse datasets to validate and expand upon our findings.

## 5. Conclusions

Adverse effects of a sedentary lifestyle on an individual’s overall health are inevitable. With reference to primary-school-aged children, the establishment of effective postural hygiene is critical as it not only promotes optimal musculoskeletal development but also significantly influences their long-term well-being and productivity. Thus, proper identification and management of contributing factors to static postural loading in this age group is essential.

Furthermore, as we advance into the era of precision occupational health, the need for highly effective and efficient assessment methods becomes increasingly significant. In various clinical settings, where the objective is to develop a model that accurately forecasts the outcome, regularized regression methods have evidently shown great performance. These models can solely be utilized to handle issues of overfitting and collinearity among predicting variables. Nevertheless, special attention is required in model selection and development, data processing, regularization, and validation phases. Eventually, this will become the art of machine learning in enhancing people’s healthy lifestyles.

## Figures and Tables

**Table 1:** Participants’ data and results of sedentary task-related measures.

Variable/Measure	Mean ± SD (Range)
Age (years)	9.4 ± 1.7 (7–12)
BMI (kg/m^2^)	16.9 ± 4.8 (7.5–28.6)
Body Fat Percentage (%)	16.5 ± 5 (8.2–30.1)
Sedentary Behavior (hours/week)	15.3 ± 2.4 (10–19)
Task Duration (minutes)	14.2 ± 3.3 (9–23)
Postural Risk (scores 1–7)	3 ± 1.2 (1–5)
	**n (%)**
Gender	
Male	130 (50.3)
Female	128 (49.7)
Musculoskeletal Health (pGALS examination)	
Healthy	101 (60.9)
Condition	157 (39.1)
Anthropometric Match (standard equations)	
Matched	120 (46.5)
Mismatched	138 (53.5)
Sitting Type	
Upright	74 (28.7)
Leaning Forward	64 (24.8)
Slouched	66 (25.6)
Slumped	54 (20.9)

**Table 2:** Results of model performance comparisons.

Metric	R^2^		MAE		RMSE	
	Train	Test	Train	Test	Train	Test
**MLR**	0.876	0.821	2.157	2.494	2.831	3.202
**EN**	0.875	0.821	2.118	2.452	2.851	3.199
n = 258 (train dataset: 206, test dataset: 52)						

**Table 3:** Results of permutation importance analysis.

MLR			EN		
Predictor	Importance	SD	Predictor	Importance	SD
Postural Risk	0.2082	0.04762	Postural Risk	0.118199	0.032799
BMI	0.075443	0.030741	Sedentary Behavior	0.107396	0.03266
Task Duration	0.063287	0.024169	BMI	0.103434	0.036781
Sedentary Behavior	0.025567	0.017667	Task Duration	0.081299	0.025873
Sitting Type— Slouched	0.002511	0.001873	Age	0.002045	0.001721
Gender— Male	0.002354	0.000987	Sitting Type— Leaning Forward	0.001049	0.002653
Gender— Female	0.002354	0.000987	Gender— Male	0.000265	0.000106
Age	0.002107	0.001306	Gender— Female	0.000265	0.000106
Sitting Type— Leaning Forward	0.001749	0.005937	Anthropometric Match—Mismatched	0.000004	0.000047
Musculoskeletal Health— Healthy	0.000054	0.000618	Anthropometric Match—Matched	0.000003	0.000045
Musculoskeletal Health— With Condition	0.000054	0.000618	Musculoskeletal Health—Healthy	0	0
Anthropometric Match—Mismatched	−0.000501	0.002352	Musculoskeletal Health—With Condition	0	0
Anthropometric Match— Matched	−0.000501	0.002352	Sitting Type— Slouched	0	0
Sitting Type— Upright	−0.001244	0.000808	Sitting Type— Upright	0	0
Body Fat Percentage	−0.011036	0.006458	Sitting Type— Slumped	−0.003859	0.001491
Sitting Type— Slumped	−0.015018	0.00631	Body Fat Percentage	−0.011674	0.008509

## Data Availability

The data presented in this study are available upon request from the corresponding author. Computer codes developed for this research are shared in GitHub and can be accessed via the following link: https://github.com/RegressionProject/Codes/blob/5c53b3095b2d409eae3f3b37b753f8a70c7e83a5/Regression_Project_Codes%20(Updated).ipynb.

## Supplementary Materials

The following supporting information can be downloaded at https://www.mdpi.com/article/10.3390/children12060744/s1, Figure S1: Cook’s distance calculated for all observations; Figure S2: Sensitivity analysis boxplot of mean imputation; Figure S3: Regularization path for the elastic net model; Figure S4: Permutation importance charts for the multiple linear regression and elastic net models; Table S1: Predictors of the static postural loading that were included in regression model development; Appendix S1: Brief description of regularized elastic model regression model.

## Abbreviations

The following abbreviations are used in this manuscript:

SPL	Static Postural Loading
MLR	Multiple Linear Regression
EN	Elastic Net
pGALS	Pediatric version of Gait, Arms, Legs, and Spine Musculoskeletal Examination
REBA	Rapid Entire Body Assessment

## References

[1] De Freitas J., Glicksberg B.S., Johnson K.W., Miotto R., Al’Aref S.J., Singh G., Baskaran L., Metaxas D. (2021). Chapter 4—Deep learning for biomedical applications. Machine Learning in Cardiovascular Medicine.

[2] Robinson H.A., Dam R., Hassan L., Jenkins D., Buchan I., Sperrin M. (2019). Post-2000 growth trajectories in children aged 4–11 years: A review and quantitative analysis. Prev. Med. Rep..

[3] Williams K., Thomson D., Seto I., Contopoulos-Ioannidis D., Ioannidis J., Curtis S., Constantin E., Batmanabane G., Hartling L., Klassen T. (2012). Standard 6: Age Groups for Pediatric Trials. Pediatrics.

[4] Tofts L., Das S., Collins F., Burton K.L. (2017). Growth charts for Australian children with achondroplasia. Am. J. Med. Genet. Part A.

[5] Weinstein S.L., Dolan L.A., Holt J. (2021). There’s More to Consider Than Thoracic Spine Height—The Case for Primary Spine Fusion in Older Early-onset Scoliosis Patients. Spine.

[6] Chen C., Chen Y., Zhang Y., Sun W., Jiang Y., Song Y., Zhu Q., Mei H., Wang X., Liu S. (2018). Association between dietary patterns and precocious puberty in children: A population-based study. Int. J. Endocrinol..

[7] Martos-Moreno G.Á., Martínez-Villanueva J., González-Leal R., Chowen J.A., Argente J. (2019). Sex, puberty, and ethnicity have a strong influence on growth and metabolic comorbidities in children and adolescents with obesity: Report on 1300 patients (the Madrid Cohort). Pediatr. Obes..

[8] Downing K.L., Salmon J., Timperio A., Hinkley T., Cliff D.P., Okely A.D., Hesketh K.D. (2019). Sitting and screen time outside school hours: Correlates in 6-to 8-year-old children. J. Phys. Act. Health.

[9] Sherry A.P., Pearson N., Ridgers N.D., Barber S.E., Bingham D.D., Nagy L.C., Clemes S.A. (2019). activPAL-measured sitting levels and patterns in 9–10 years old children from a UK city. J. Public Health.

[10] Reilly J.J., Barnes J., Gonzalez S., Huang W.Y., Manyanga T., Tanaka C., Tremblay M.S. (2022). Recent Secular Trends in Child and Adolescent Physical Activity and Sedentary Behavior Internationally: Analyses of Active Healthy Kids Global Alliance Global Matrices 1.0 to 4.0. J. Phys. Act. Health.

[11] Abbass S.J., Abd-ul-Amir D.Q. (2017). Effects of Backpack Loads on Kids Posture. Al-Nahrain J. Eng. Sci..

[12] Brzęk A., Dworrak T., Strauss M., Sanchis-Gomar F., Sabbah I., Dworrak B., Leischik R. (2017). The weight of pupils’ schoolbags in early school age and its influence on body posture. BMC Musculoskelet. Disord..

[13] Ihnow S.B., Sturm P.F. (2021). Case of an Adolescent Male with Back Pain and Poor Posture: Scheuermann’s Kyphosis. Back Pain in the Young Child and Adolescent.

[14] Kumar P., Singh R.M., Ratnakar A. (2018). Role of physical education research activities and their impact in modern day life. Asian J. Multidimens. Res. (AJMR).

[15] Karalus M.A., Sullivan T.A., Wild C.E., Cave T.L., O’Sullivan N.A., Hofman P.L., Edwards E.A., Mouat S., Wong W., Anderson Y.C. (2021). The cost of investigating weight-related comorbidities in children and adolescents in Aotearoa/New Zealand. J. Paediatr. Child Health.

[16] Li X., Vanderloo L.M., Maguire J.L., Keown-Stoneman C.D.G., Aglipay M., Anderson L.N., Cost K.T., Charach A., Vanderhout S.M., Birken C.S. (2021). Public health preventive measures and child health behaviours during COVID-19: A cohort study. Can. J. Public Health.

[17] Neil A.L., Islam F., Kariuki M., Laurens K.R., Katz I., Harris F., Carr V.J., Green M.J. (2020). Costs for physical and mental health hospitalizations in the first 13 years of life among children engaged with Child Protection Services. Child Abus. Negl..

[18] Raudenbush B.L., Gurd D.P., Goodwin R.C., Kuivila T.E., Ballock R.T. (2017). Cost analysis of adolescent idiopathic scoliosis surgery: Early discharge decreases hospital costs much less than intraoperative variables under the control of the surgeon. J. Spine Surg..

[19] Wolfe I., Satherley R.-M., Scotney E., Newham J., Lingam R. (2020). Integrated care models and child health: A meta-analysis. Pediatrics.

[20] Zeng W., Li G., Ahn H., Nguyen H.T.H., Shepard D.S., Nair D. (2018). Cost-Effectiveness of health systems strengthening interventions in improving maternal and child health in low-and middle-income countries: A systematic review. Health Policy Plan..

[21] Hogan M.E., Taddio A., Katz J., Shah V., Krahn M. (2016). Incremental health care costs for chronic pain in Ontario, Canada: A population-based matched cohort study of adolescents and adults using administrative data. Pain.

[22] Bevan S. (2015). Economic impact of musculoskeletal disorders (MSDs) on work in Europe. Best Pract. Res. Clin. Rheumatol..

[23] WHO (2021). Health Promotion and Disease Prevention Through Population-Based Interventions.

[24] Hulshof C.T., Pega F., Neupane S., van der Molen H.F., Colosio C., Daams J.G., Descatha A., Kc P., Kuijer P.P., Mandic-Rajcevic S. (2021). The prevalence of occupational exposure to ergonomic risk factors: A systematic review and meta-analysis from the WHO/ILO Joint Estimates of the Work-related Burden of Disease and Injury. Environ. Int..

[25] OSHA, EU (2019). EU-OSHA—European Agency for Safety and Health at Work, Work-Related Musculoskeletal Disorders: Prevalence, Costs and Demographics in the EU. 2019, EU-OSHA.

[26] Wu H.-C., Wang M.-J.J. (2002). Relationship between maximum acceptable work time and physical workload. Ergonomics.

[27] Aulia S.F., Alayyannur P.A., Dwiyanti E., Martiana T., Arini S.Y. (2021). Correlation between Workload and Work Environment with Work Stress. Indian J. Forensic Med. Toxicol..

[28] DiDomenico A., Nussbaum M.A. (2011). Effects of different physical workload parameters on mental workload and performance. Int. J. Ind. Ergon..

[29] Schwartz A., Gerberich S.G., Albin T., Kim H., Ryan A.D., Church T.R., Green D.R., McGovern P.M., Erdman A.G., Arauz R.F. (2020). The association between janitor physical workload, mental workload, and stress: The SWEEP study. Work.

[30] Oh J.-I., Yoo D.-H., Paek D.-M., Park J.-S., Cho S.-I. (2011). Association between physical workload and work-related back pain: A Nationwide study. Korean J. Occup. Environ. Med..

[31] Hashiguchi N., Kodama K., Lim Y., Che C., Kuroishi S., Miyazaki Y., Kobayashi T., Kitahara S., Tateyama K. (2020). Practical judgment of workload based on physical activity, work conditions, and worker’s age in construction site. Sensors.

[32] Jørgensen M.B., Nabe-Nielsen K., Clausen T., Holtermann A. (2013). Independent effect of physical workload and childhood socioeconomic status on low back pain among health care workers in Denmark. Spine.

[33] Mukherjee S., Rifkin R., Poggio T., Denison D.D., Hansen M.H., Holmes C.C., Mallick B., Yu B. (2003). Regression and Classification with Regularization. Nonlinear Estimation and Classification.

[34] Bray A. (2016). Evaluation of Human Work.

[35] Joshi Mangesh D.V. (2019). A systematic review of comparative studies on ergonomic assessment techniques. Int. J. Ind. Ergon..

[36] Hedge A., Morimoto S., McCrobie D.J.E. (1999). Cornell musculoskeletal discomfort questionnaire. Ergonomics.

[37] Osqueizadeh R., Bandpei M.A.M., Rahmani N., Goudarzi H., Ebadi A. (2023). Reliability and Validity of Observational Methods for Postural Load Assessment: An Updated Systematic Review. Health Scope.

[38] Young N.L., Williams J.I., Yoshida K.K., Wright J.G. (2000). Measurement properties of the activities scale for kids. J. Clin. Epidemiol..

[39] lisahunter, Abbott R., Macdonald D., Ziviani J., Cuskelly M. (2014). Active kids active minds: A physical activity intervention to promote learning?. Asia-Pac. J. Health Sport Phys. Educ..

[40] Aittasalo M., Jussila A., Tokola K., Sievänen H., Vähä-Ypyä H., Vasankari T. (2019). Kids Out; evaluation of a brief multimodal cluster randomized intervention integrated in health education lessons to increase physical activity and reduce sedentary behavior among eighth graders. BMC Public Health.

[41] Nogueira R.C., Weeks B.K., Beck B.R. (2014). An in-school exercise intervention to enhance bone and reduce fat in girls: The CAPO Kids trial. Bone.

[42] Rosenberg D.E., Norman G.J., Wagner N., Patrick K., Calfas K.J., Sallis J.F. (2010). Reliability and Validity of the Sedentary Behavior Questionnaire (SBQ) for Adults. J. Phys. Act. Health.

[43] Bost K., Teran-Garcia M., Donovan S., Fiese B., Team S.K. (2018). Child body mass index, genotype and parenting in the prediction of restrictive feeding. Pediatr. Obes..

[44] Li X., Keown-Stoneman C.D., Lebovic G., Omand J.A., Adeli K., Hamilton J.K., Hanley A.J., Mamdani M., McCrindle B.W., Sievenpiper J.L. (2020). The association between body mass index trajectories and cardiometabolic risk in young children. Pediatr. Obes..

[45] Birch L., Perry R., Hunt L.P., Matson R., Chong A., Beynon R., Shield J.P. (2019). What change in body mass index is associated with improvement in percentage body fat in childhood obesity? A meta-regression. BMJ Open.

[46] Elliott S.A., Truby H., Lee A., Harper C., Abbott R.A., Davies P.S. (2011). Associations of body mass index and waist circumference with: Energy intake and percentage energy from macronutrients, in a cohort of Australian children. Nutr. J..

[47] Weststrate J.A., Deurenberg P. (1989). Body composition in children: Proposal for a method for calculating body fat percentage from total body density or skinfold-thickness measurements. Am. J. Clin. Nutr..

[48] Nogueira R.C., Weeks B.K., Beck B.R. (2015). Characterisation of the mechanical loads and metabolic intensity of the CAPO kids exercise intervention for healthy primary school children. J. Sports Sci. Med..

[49] Naser S.S.A., Al-Bayed M.H. (2016). Detecting Health Problems Related to Addiction of Video Game Playing Using an Expert System. World Wide J. Multidiscip. Res. Dev..

[50] Foster H.E., Jandial S. (2013). pGALS–paediatric Gait Arms Legs and Spine: A simple examination of the musculoskeletal system. Pediatr. Rheumatol..

[51] Xue Z., Li Q., Ashraf M. (2023). Designing wearables for assistive correction of children’s sitting posture. Machine Learning, Multi Agent and Cyber Physical Systems: Proceedings of the 15th International FLINS Conference (FLINS 2022).

[52] Sahabo M., Kabara A. (2023). Anthropometrics and ergonomics of secondary school students in four yola metropolis, Adamawa State, Nigeria. J. Appl. Sci. Environ. Manag..

[53] Castellucci H., Arezes P., Molenbroek J. (2015). Equations for defining the mismatch between students and school furniture: A systematic review. Int. J. Ind. Ergon..

[54] Aminian S., Hinckson E.A., Stewart T. (2015). Modifying the classroom environment to increase standing and reduce sitting. Build. Res. Inf..

[55] Kimmerly L., Odell D. (2009). Children and computer use in the home: Workstations, behaviors and parental attitudes. Work.

[56] Sedrez J.A., Rosa M.I.Z.d., Noll M., Medeiros F.d.S., Candotti C.T. (2015). Risk factors associated with structural postural changes in the spinal column of children and adolescents. Rev. Paul. De Pediatr..

[57] McAtamney L., Hignett S. (2004). Rapid entire body assessment. Handbook of Human Factors and Ergonomics Methods.

[58] O’Brien R.M. (2017). Dropping highly collinear variables from a model: Why it typically is not a good idea. Soc. Sci. Q..

[59] Zou H., Hastie T. (2005). Regularization and variable selection via the elastic net. J. R. Stat. Soc. Ser. B Stat. Methodol..

[60] Altmann A., Toloşi L., Sander O., Lengauer T. (2010). Permutation importance: A corrected feature importance measure. Bioinformatics.

[61] Software P.P. www.python.org.

[62] Menor-Rodríguez M.J., Rodríguez-Blanque R., Montiel-Troya M., Cortés-Martín J., Aguilar-Cordero M.J., Sánchez-García J.C. (2022). Educational Intervention in the Postural Hygiene of School-Age Children. Healthcare.

[63] Balkó S., Balkó I., Valter L., Jelínek M. (2017). Influence of physical activities on the posture in 10-11 year old schoolchildren. J. Phys. Educ. Sport.

[64] Hellig T., Mertens A., Brandl C. (2018). The interaction effect of working postures on muscle activity and subjective discomfort during static working postures and its correlation with OWAS. Int. J. Ind. Ergon..

[65] Warda D.G., Nwakibu U., Nourbakhsh A. (2023). Neck and Upper Extremity Musculoskeletal Symptoms Secondary to Maladaptive Postures Caused by Cell Phones and Backpacks in School-Aged Children and Adolescents. Healthcare.

[66] Tanaka C., Tanaka M., Tanaka S. (2018). Objectively evaluated physical activity and sedentary time in primary school children by gender, grade and types of physical education lessons. BMC Public Health.

[67] Ellis Y.G., Cliff D.P., Janssen X., Jones R.A., Reilly J.J., Okely A.D. (2017). Sedentary time, physical activity and compliance with IOM recommendations in young children at childcare. Prev. Med. Rep..

[68] McLellan G., Arthur R., Donnelly S., Buchan D.S. (2020). Segmented sedentary time and physical activity patterns throughout the week from wrist-worn ActiGraph GT3X+ accelerometers among children 7–12 years old. J. Sport Health Sci..

[69] Hidding L.M., Altenburg T.M., van Ekris E., Chinapaw M.J.M. (2017). Why Do Children Engage in Sedentary Behavior? Child- and Parent-Perceived Determinants. Int. J. Environ. Res. Public Health.

[70] Enright G., Allman-Farinelli M., Redfern J. (2020). Effectiveness of family-based behavior change interventions on obesity-related behavior change in children: A realist synthesis. Int. J. Environ. Res. Public Health.

[71] McLaughlin T.W., Denney M.K., Snyder P.A., Welsh J.L. (2012). Behavior support interventions implemented by families of young children: Examination of contextual fit. J. Posit. Behav. Interv..

[72] Saunders T.J., Rollo S., Kuzik N., Demchenko I., Bélanger S., Brisson-Boivin K., Carson V., da Costa B.G.G., Davis M., Hornby S. (2022). International school-related sedentary behaviour recommendations for children and youth. Int. J. Behav. Nutr. Phys. Act..

[73] Guirado T., Chambonnière C., Chaput J.-P., Metz L., Thivel D., Duclos M. (2021). Effects of Classroom Active Desks on Children and Adolescents’ Physical Activity, Sedentary Behavior, Academic Achievements and Overall Health: A Systematic Review. Int. J. Environ. Res. Public Health.

[74] Hnatiuk J.A., Salmon J., Hinkley T., Okely A.D., Trost S. (2014). A review of preschool children’s physical activity and sedentary time using objective measures. Am. J. Prev. Med..

[75] Phillips S.M., Summerbell C., Hobbs M., Hesketh K.R., Saxena S., Muir C., Hillier-Brown F.C. (2021). A systematic review of the validity, reliability, and feasibility of measurement tools used to assess the physical activity and sedentary behaviour of pre-school aged children. Int. J. Behav. Nutr. Phys. Act..

[76] Dowda M., Saunders R.P., Dishman R.K., Pate R.R. (2024). Association of physical activity, sedentary behavior, diet quality with adiposity: A longitudinal analysis in children categorized by baseline weight status. Int. J. Obes..

[77] Maciałczyk-Paprocka K., Stawińska-Witoszyńska B., Kotwicki T., Sowińska A., Krzyżaniak A., Walkowiak J., Krzywińska-Wiewiorowska M. (2017). Prevalence of incorrect body posture in children and adolescents with overweight and obesity. Eur. J. Pediatr..

