# Supplementary material for: Predictors of Static Postural Loading in Primary-School-Aged Children: Comparing Elastic Net and Multiple Regression Methods
Source: Children (Basel). 2025 Jun 8;12(6):744. doi: 10.3390/children12060744 (PMC12191867; doi:10.3390/children12060744)
Supplement: Supplementary file 1 [file children-12-00744-s001.zip › children-3497692-supplementary.pdf]

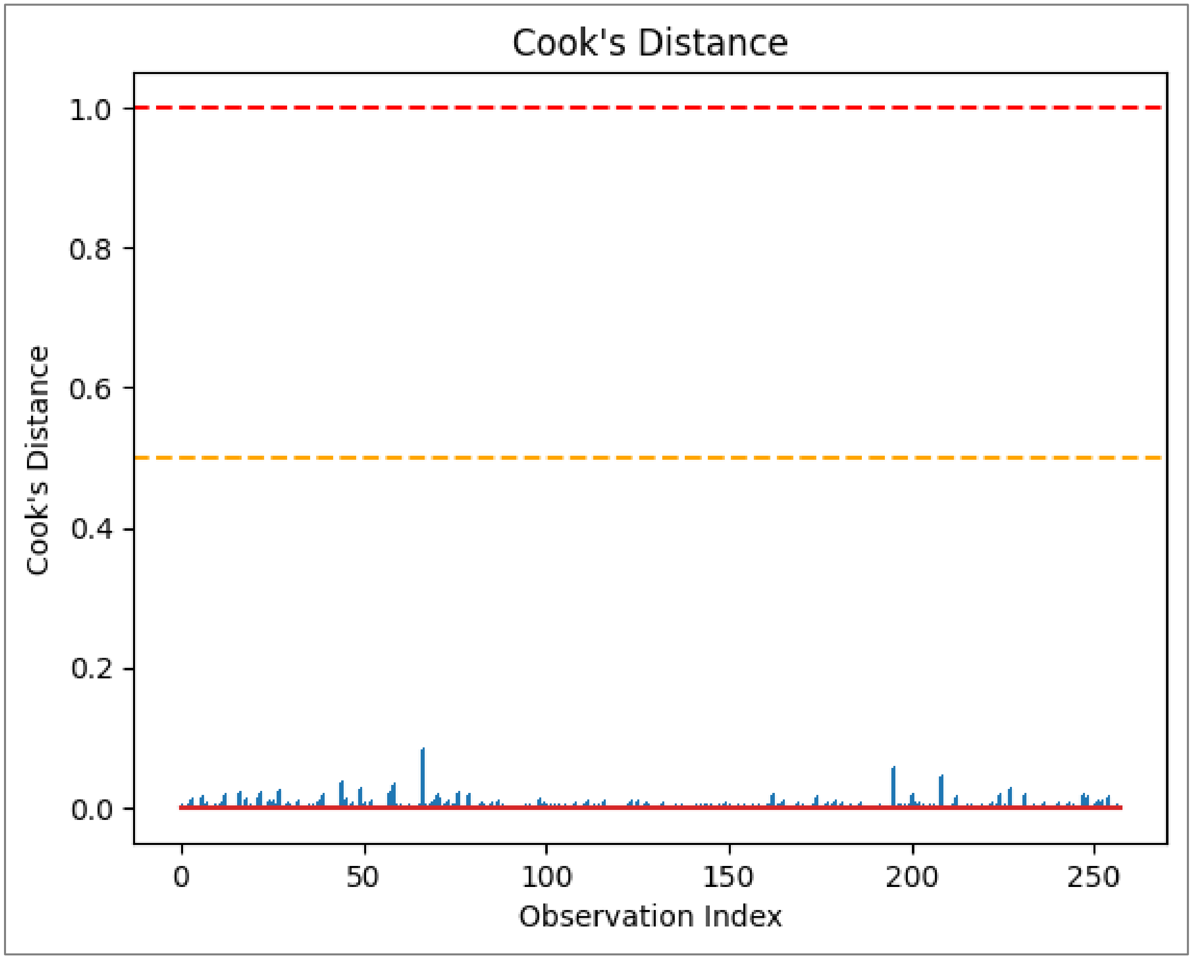

Figure S1. Cook's Distance Calculated for All Observations. The plot was generated by Matplotlib library in Python (Version 3-12-2).

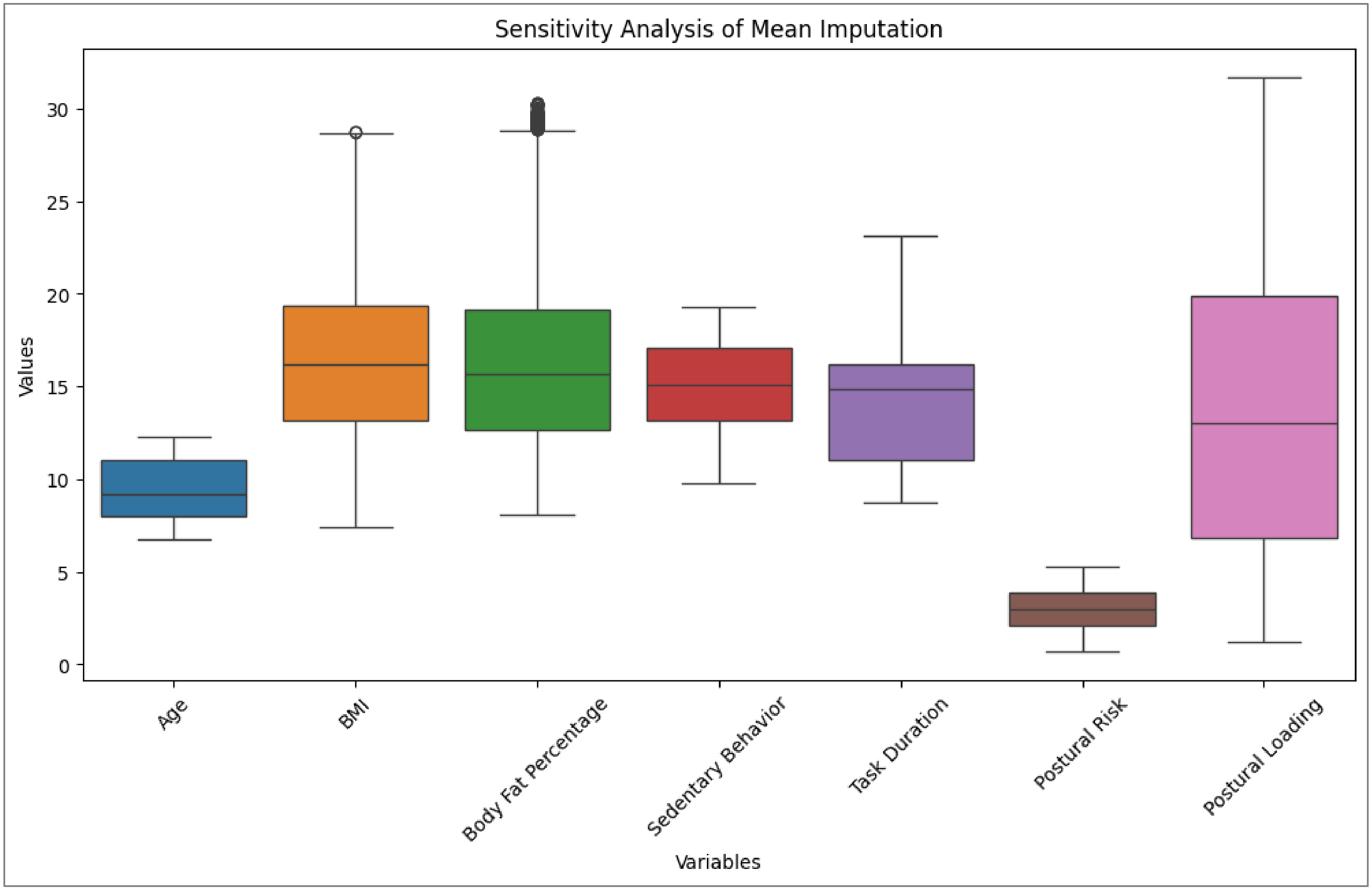

Figure S2. Sensitivity Analysis Boxplot of the Mean Imputation. The plot was generated by Matplotlib library in Python (Version 3-12-2).

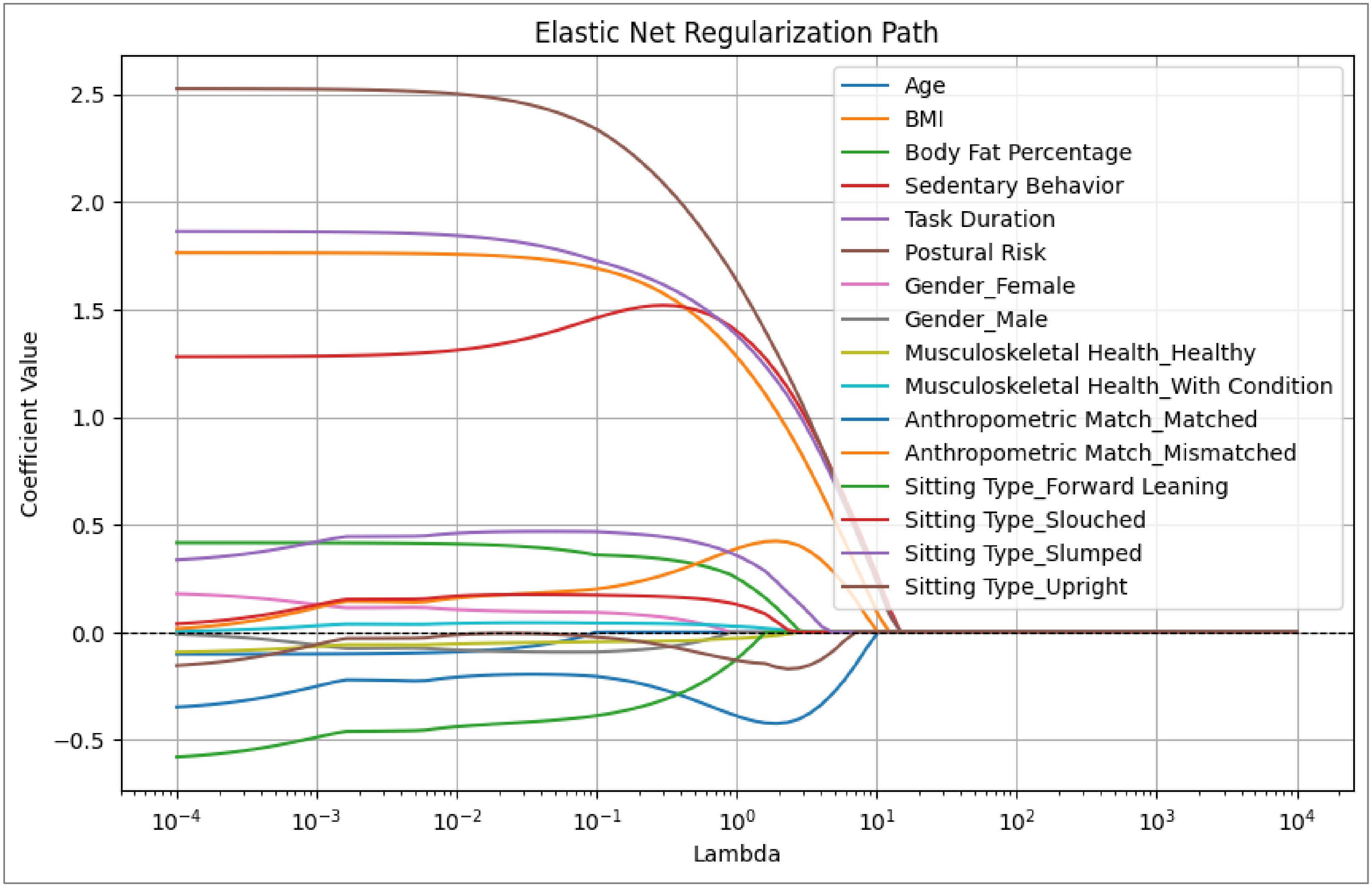

Figure S3. Regularization Path for the Elastic Net Model. The plot was generated by Matplotlib library in Python (Version 3-12-2). The optimal alpha value for model performance was 0.17.

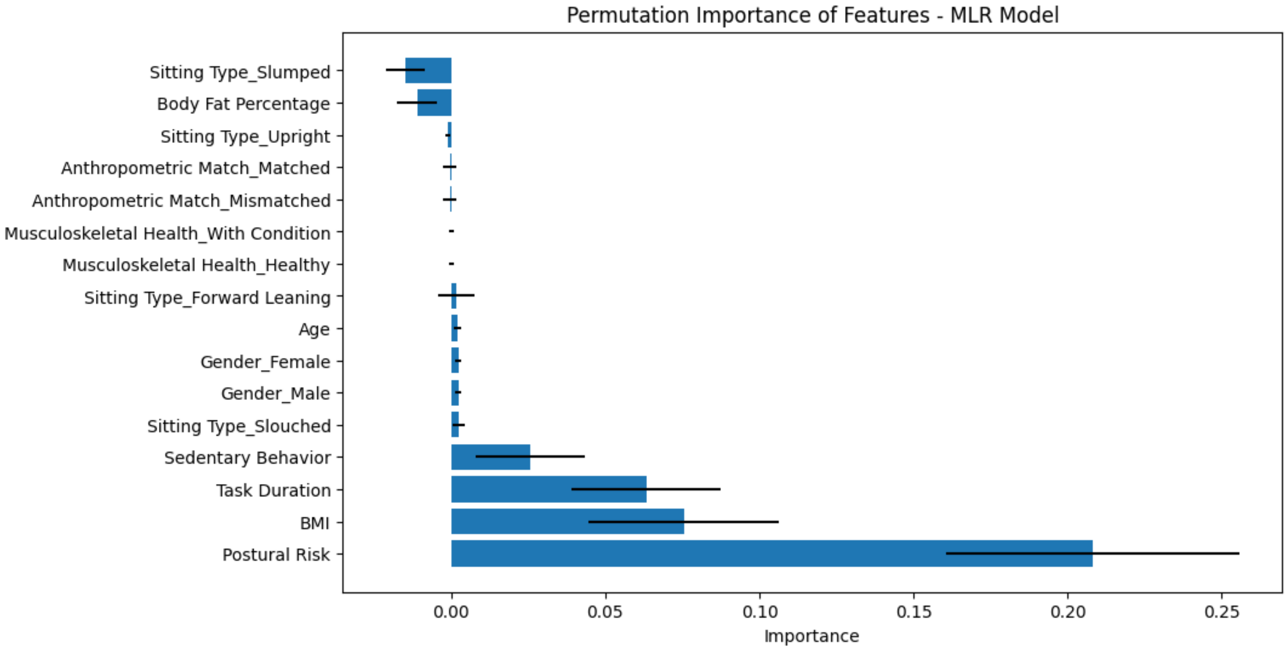

(a)

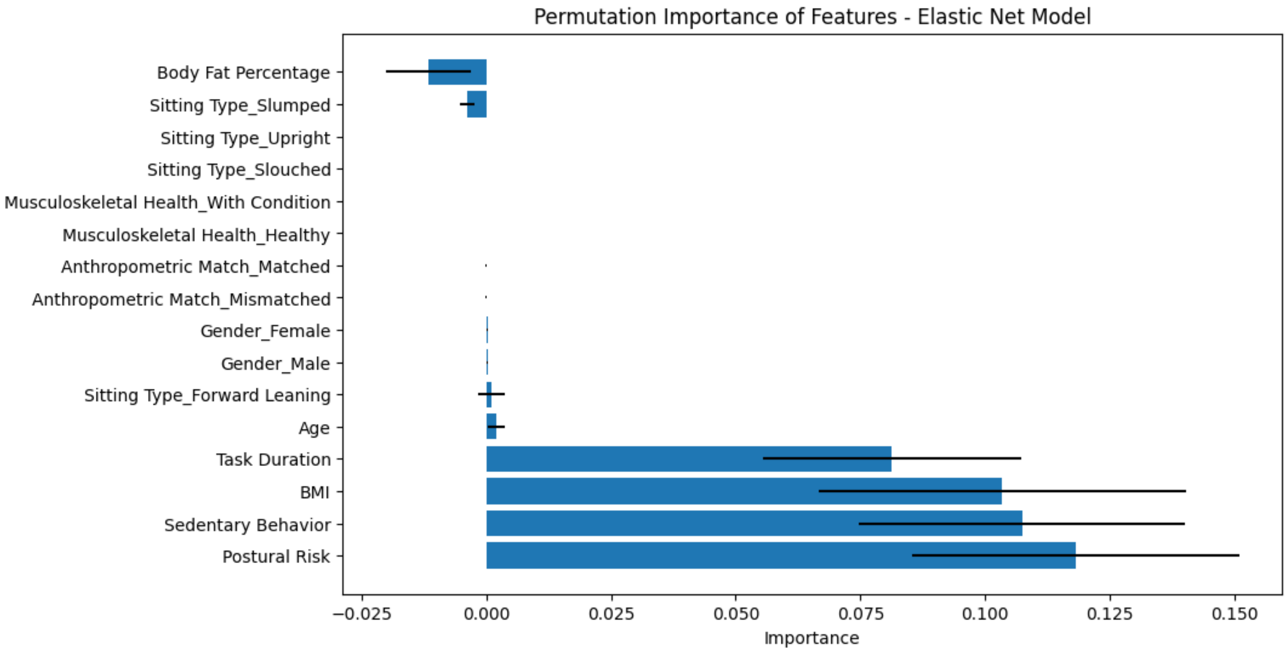

(b)

Figure S4. Permutation Importance Plots for the Multiple Linear Regression (a) and the Elastic Net (b) Models. The optimal alpha value for model performance was 0.17. The plots were generated by Matplotlib library in Python (Version 3-12-2).

Table S1. Predictors of the Static Postural Loading that were included in Regression Model Development

Predictor	Description	Assessment	Unit/ Categories
**Age**	The period contemporary with a person's lifetime or with his or her active life	Demographics form	Years
**Gender**	The biological and physiological characteristics that define humans as female or male.	Individual's biological sex (female/male)	Female/Male
**BMI**	The metric for defining anthropometric height/weight characteristics in adults, and for classifying (categorizing) them into group.	Calculated according to individual's weight and height	kg/m^2
**Body Fat Percentage**	Distribution of fat, muscle, bone, and other tissues that make up individual's body	Calculated according to four-site Westrarte Equation	%
**Sedentary Behavior**	Any waking behavior characterized by an energy expenditure ≤1.5 metabolic equivalents, while in a sitting, reclining, or lying posture	Sedentary Behaviour Questionnaire (SBQ)	Hours
**Task Duration**	Time span during which a static task is performed	Duration of being engaged in the sedentary task	Minutes
**Postural Risk**	Physical risk imposed on individual's body while being in a specific body position	Rapid Entire Body Assessment (REBA) semi-quantitative tool (1-8)	1-8
**Musculoskeletal Health**	Function and appearance of the Musculoskeletal system	Results of pediatric Gait, Arms, Legs, Spine (pGALS) assessment checklist	Healthy/With Condition
**Anthropometric Match**	Match between sitting furniture and specific body dimensions	Chair Seat Height vs Popliteal Height<br>Chair Seat Depth vs Buttock-Popliteal Length Chair Seat Width vs Pelvic Width<br>Upper Edge of Backrest vs Sitting Shoulder Height<br>Desk Height vs Sitting Elbow Height	Matched/Mismatched
**Sitting Type**	Position of the body while sitting	Direct observation	Upright<br>Leaning forward<br>Slumped<br>Slouched

Appendix S1. Brief Description of the Regularized Elastic Model Regression Model.

Elastic Net (EN) combines the strengths of both LASSO and Ridge regularized regressions to control multicollinearity issues. The mathematical representation of EN requires the minimization of the loss function presented in the following equation:

$$J(\beta) = \frac{1}{2n}\sum_{i=1}^{n}\left(\gamma_i - X_i\beta\right)^2 + \lambda\left(\alpha\sum_{j=1}^{p}\left|\beta_j\right| + \left(\frac{1-\alpha}{2}\right)\sum_{j=1}^{p}{\beta_j}^2\right)$$

Where, $J(\beta)$ is the objective function to be minimized, γ_i is the response variable for observation i, X_i is the vector of predictor variables for observation i, β is the vector of coefficiens to be estimated, λ Is the ovrall regularization strength, α is the mixing parameter that controls the balance between LASSO and Ridge penalties, n is the number of observations, and p is the number of preditors. If $\alpha = 1$, the model behaves like LASSO regression, and L1 penalty $\left(\sum_{j=1}^{p}\left|\beta_j\right|\right)$ encourages sparsity by driving some coefficients to zero. If $\alpha = 0$, the model behaves like Ridge regression, and L2 penalty $\left(\sum_{j=1}^{p}{\beta_j}^2\right)$ helps to stabilize the estimates. In Elastic Net, where $0 < \alpha < 1$ the model utilizes both penalties. Besides, when $\lambda = 0$, the equation is left with ordinary least squares (OLS) function, creating a standard multiple linear regression (MLR) model. As it increases, the coefficients shrink, and ultimately, when λ is set to a large value, the model effectively shrinks the coefficients of the predictor variables towards zero. As a result, only the intercept may remain significantly non-zero, leading to a model that primarily captures the mean of the response variable, while disregarding the contributions of the predictors.
